# Supplementary figures and images for: Novel method for combining microbial bioremediation with static magnetic fields to remediate mercury-contaminated soils
Source: PLoS One. 2025 Aug 22;20(8):e0330872. doi: 10.1371/journal.pone.0330872 (PMC12373231; doi:10.1371/journal.pone.0330872)

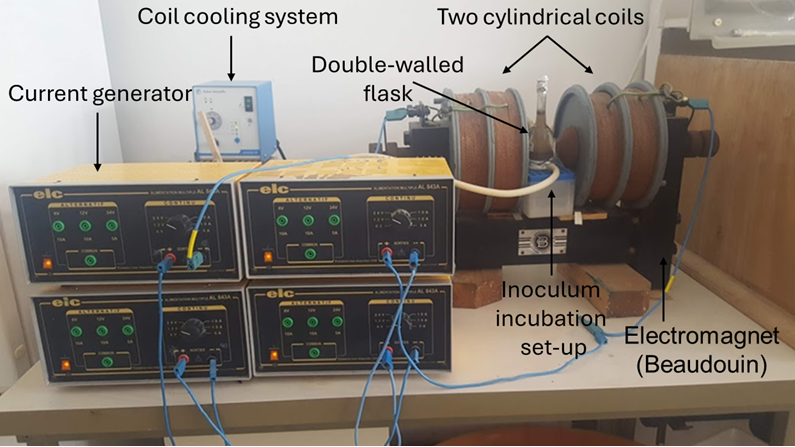

Supplement: S1 Fig — (TIF) [file pone.0330872.s001.tif]
