# Supplementary material for: Novel method for combining microbial bioremediation with static magnetic fields to remediate mercury-contaminated soils
Source: PLoS One. 2025 Aug 22;20(8):e0330872. doi: 10.1371/journal.pone.0330872 (PMC12373231; doi:10.1371/journal.pone.0330872)
Supplement: S1 Table — (PDF) [file pone.0330872.s002.pdf]

S1_Table : Detection end quantification limits of the target Elements (ICP-OES).

Element	LOD (mg/Kg)	LOQ (mg/Kg)
Cadmium (Cd)	0.05	0.15
Cobalt (Co)	0.1	0.25
Copper (Cu)	0.1	0.3
Iron (Fe)	0.5	1.5
Lead (Pb)	0.2	0.6
Manganese (Mn)	0.1	0.3
Nickel (Ni)	0.2	0.6
Zinc (Zn)	0.2	0.6
Chromium (Cr)	0.1	0.3
Calcium (Ca)	0.2	0.48
Magnesium (Mg)	0.1	0.25
Sodium (Na)	0.1	0.6
Potassium (K)	0.1	0.86
Phosphorus (P)	0.1	0.5

LOD: Limit of detection; LOQ: Limit of quantification
