# Supplementary material for: Novel method for combining microbial bioremediation with static magnetic fields to remediate mercury-contaminated soils
Source: PLoS One. 2025 Aug 22;20(8):e0330872. doi: 10.1371/journal.pone.0330872 (PMC12373231; doi:10.1371/journal.pone.0330872)
Supplement: Attachment — Submitted filename: Peer Review Report for PONE.pdf [file pone.0330872.s006.pdf]

Peer Review Report for PONE-D-24-56633

Manuscript Title: Novel method for combining microbial bioremediation with static magnetic fields to remediate mercury-contaminated soils

---

Technical Quality

1. **Page 2, Lines 33–39**:
   - **Issue**: The abstract states remediation rates of 49.36% (non-sterile) and 72.49% (sterile) with SMF, but the Results section (Page 10–12) reports 81.77 and 87.17%, respectively. Inconsistency in key metrics undermines reliability.
   - **Recommendation**: Reconcile values throughout the manuscript. Clearly define how "remediation acceleration" (%) is calculated (e.g., vs. control).
2. **Page 7, Lines 133–141**:
   - **Issue**: Incomplete description of controls. Flasks 3 and 4 are labeled as non-sterile soil + M9 but lack clarity on whether they include *P. stutzeri* LBR.
   - **Recommendation**: Clarify experimental groups (e.g., "Flask 3: Non-sterile soil + M9 [no bacteria]").
3. **Page 12, Lines 242–245**:
   - **Issue**: Attribution of bacterial decline to pyruvate depletion is speculative without supporting data (e.g., pyruvate measurements).
   - **Recommendation**: Provide data on carbon source depletion or temper conclusions.
4. **Page 15, Lines 331–334**:
   - **Issue**: Proposed mechanism (SMF enhancing MerA activity) is unsupported by experimental evidence (e.g., enzyme assays).
   - **Recommendation**: Include data on MerA activity under SMF or reframe as a hypothesis.

Language & Clarity

1. **Throughout**:
   - **Issue**: Inconsistent italicization of bacterial names (e.g., *Pseudomonas stutzeri* vs. Pseudomonas stutzeri).
   - **Recommendation**: Italicize all genus/species names (e.g., *P. stutzeri* LBR).

2. **Page 3, Line 64**:
   - **Issue**: "borne on plasmids" → should be "born on plasmids."
   - **Correction**: Replace with "born."
3. **Page 10, Lines 210–214**:
   - **Issue**: Redundant phrasing ("non-bioaugmented non-sterile soil").
   - **Correction**: Simplify to "non-bioaugmented soil."
4. **Page 13, Lines 268–269**:
   - **Issue**: Awkward syntax ("deployment of SMF in environmental engineering with the objective of...").
   - **Correction**: "We deployed SMF in environmental engineering to enhance soil mercury remediation."

Reporting & Methodology

1. **Page 6, Line 118**:
   - **Issue**: Ambiguous mercury-doping concentration ("148 mg $HgSO_4$ diluted in 100 mL"). Final soil Hg concentration unclear.
   - **Recommendation**: Specify Hg concentration in soil post-doping (mg/kg).
2. **Page 9, Table 1**:
   - **Issue**: Units for Iron (6504 mg/kg) and Chromium (341.03 mg/kg) suggest potential contamination, yet unaddressed.
   - **Recommendation**: Comment on background metal levels' relevance to Hg remediation.
3. **Page 14, Lines 298–302**:
   - **Issue**: Bacterial counts use "10°" (e.g., 27 x 10° CFU/mL) instead of "10^{9}".
   - **Correction**: Replace "10°" with "10^{9}" throughout.
4. **Page 16, Line 345**:
   - **Issue**: Acknowledgments list collaborators but omit funding sources.
   - **Recommendation**: Declare funding or state "No funding was received."

Data Availability

1. **Throughout**:
   - **Issue**: No statement on data repository accession for raw data (e.g., bacterial counts, Hg measurements).
   - **Recommendation**: Add a *Data Availability* section per PLOS ONE policy (e.g., "Data are available at DOI: [link]").
2. **Page 6, Line 125**:
   - **Issue**: GenBank accession number for *P. stutzeri* LBR sequences is unreported.
   - **Recommendation**: Provide accession number in *Methods*.

Summary

The study presents a novel approach with significant potential. However, **major revisions** are required to address:

- Technical inconsistencies (remediation rates, mechanism support).
- Clarification of methods/controls.
- Compliance with data-sharing policies.
- Language/formatting corrections.

Recommendation: **Revise**

Peer Review Comments

Page	Line(s)	Section	Issue	Recommendation
2	33–39	Abstract	Inconsistent remediation rates (49.36% in Abstract vs. 81.77%/87.17% in Results).	Reconcile values; define "remediation acceleration" calculation (e.g., vs. control).
7	133–141	Methods	Unclear description of control groups (Flasks 3–4).	Specify contents (e.g., "Flask 3: Non-sterile soil + M9 [no bacteria]").
12	242–245	Results	Bacterial decline attributed to pyruvate depletion without supporting data.	Provide pyruvate measurements or temper conclusions.

15	331–334	Discussion	Proposed mechanism (SMF enhances MerA activity) lacks experimental evidence.	Include MerA enzyme assay data or reframe as a hypothesis.
*	*	Language	Inconsistent italicization of bacterial names (e.g., *Pseudomonas stutzeri* vs. Pseudomonas stutzeri).	Italicize all genus/species names consistently (e.g., *P. stutzeri* LBR).
3	64	Introduction	Typo: "borne" should be "born."	Correct to "born."
10	210–214	Results	Redundant phrasing ("non-bioaugmented non-sterile soil").	Simplify to "non-bioaugmented soil."
6	118	Methods	Ambiguous mercury-doping concentration.	Specify final soil Hg concentration (mg/kg).
9	Table 1	Results	Elevated Fe (6504 mg/kg) and Cr (341.03 mg/kg) levels unaddressed.	Discuss relevance of background metals to Hg remediation.
14	298–302	Results	Bacterial counts use "10°" instead of "10^{9}" (e.g., 27 × 10° CFU/mL).	Replace "10°" with "10^{9}" throughout.
16	345	Acknowledgments	Funding source(s) not declared.	Add funding statement (e.g., "Funded by [Agency]" or "No funding received").
*	*	Data Availability	No repository statement for raw data (bacterial counts, Hg measurements).	Add section: "Data available at [DOI/repository link]."
6	125	Methods	GenBank accession number for *P. stutzeri* LBR sequences unreported.	Provide accession number in *Methods*.

Notes:

Reviewer: Dr. Mutiu A. ALABI
Confidential Competing Interests: None declared.
