# Supplementary material for: Novel method for combining microbial bioremediation with static magnetic fields to remediate mercury-contaminated soils
Source: PLoS One. 2025 Aug 22;20(8):e0330872. doi: 10.1371/journal.pone.0330872 (PMC12373231; doi:10.1371/journal.pone.0330872)
Supplement: Attachment — Submitted filename: Comments to the Editor.docx [file pone.0330872.s003.docx]

Dear Editor,

Here I send my review of the research paper entitled ‘Novel method for combining microbial bioremediation with static magnetic fields to remediate mercury-contaminated Soils’, by Naima Werfelli; Mariem Taboubi; Sirine Ridene; Ahlem Mansouri; Ahmed Landoulsi; Chiraz Abbes, (Manuscript #: PONE-D-24-56633).

The main objective of the article was to evaluate the effect of *Pseudomonas stutzeri* LBR in combination with the application of static magnetic fields (SMF) on mercury remediation. While the main results presented by the authors (in *Abstract* and *Results* sections) are encouraging, I consider that the paper needs a deep restructuring, expansion, and clarification of certain sections.

In particular, the *Introduction* section lacks information relevant to the topic of interest, delimitation of the problematic, concrete background on the bioremediation efficacy of the selected bacterial isolate (*P. stutzeri*) and of the SMF technique, presented as a novel aspect of the work. It should also be expanded and clarified which aspects of such problematic were intended to be addressed by this work and for the treatment of which type of contaminated matrices.

The *Materials and Methods* section should be rewritten, providing in detail all the information that would allow an understanding and potential repetition by any other researcher accessing the work (explaining why the selection of the sampled area, history of land use - whether it was tilled, detailed circumscription providing coordinates, numbers of samples taken, extent of the area sampled, etc.). All subsections of this section lack details, necessary for the understanding and potential repetition of the work by other researchers. The lack of detail, description and clarity of this section prevents me from consistently analyze the results presented.

Approximately 50% of the references cited in the article correspond to works that are 10 years old or more. For some statements this point is not of great relevance, but to enrich the overview, the potential and quality of the work, discussions on the subject matter addressed and the contextualization of the results obtained, it is necessary to update the bibliography.

According to the Editorial criteria highlighted as relevant for publication, I would say that those related to number 3 (description of experiments, analysis, technical standards); number 4 (clear conclusions supported by what has been presented above) and particularly number 5 (intelligible presentation of the work) are NOT met in the present work. For all the above reasons I consider that in its present form this manuscript cannot be accepted for publication.

I hope that my suggestions will help the authors to improve the work and the publisher to make a fair and informed decision in relation to the submitted work.

**Specific comments to the authors**

The writing could benefit from clearer organization. Some sections, e.g. materials and methods, lack clarity, descriptions, and smooth transitions, making it difficult for readers to follow the work done. The study should provide more details on the experimental design, including controls and replicates. This information is crucial for assessing the reliability and validity of the results. The methods of statistical analysis used to evaluate the data should be clearly described. Without this, it is challenging to assess the robustness of the conclusions drawn from the results (specific tests, data transformation, etc.). There should be a stronger connection between the *Materials and methods* and the *Results* sections. For instance, specific details about how the treatments were applied should directly correlate with the results presented. Despite the promising findings presented, the work needs to be deeply revised, addressing these suggestions could enhance the clarity, rigor, and impact of the research.

**Abstract**:

1. I suggest revising the spelling and formatting, respecting the spacing throughout the text.

2. Do not use abbreviations, particularly in the case of bacterial isolate nomenclature.

3.	When starting to refer to the results obtained, emphasize the observed effect (increase, decrease, etc.) on Hg removal instead of ‘The results demonstrated the impact of SMF on...’.

4.	The statement that the combination of SMF and *P. stutzeri* ‘accelerated’ metal remediation in sterile soil, compared to SMF alone, suggests that the effect was observed in a shorter treatment time. If this was the case, I suggest clarification, otherwise ‘accelerated’ should be replaced by increased/promoted removal by x %, relative to SMF alone.

**Introduction**:

I suggest a clear delimitation of the impact of the problematic addressed, an expansion of previous studies (e.g. on the presence of resistance genes in the bacterial isolate used, use of SMF and their main results). I suggest reorganizing the section, including a bibliographical update and finally detailing clearly how the present article aims to contribute to solving the problem in question (mentioned vaguely in lines 75-76, 81-82 and 93-94). Additionally, data should be provided on the Hg levels (ppm, mg/kg) permitted/suggested by any regulatory or health control organism.

In particular:

1- Line 42, where it is argued that mercury is a bioaccumulative ‘toxin’, I suggest revision of the term. Hg can be classified as a ‘toxic’ metal, but not a toxin, which presupposes a biological origin of the compound in question. The above-mentioned citation of reference [1] does not claim that it is a toxin. I suggest adding its classification according to regulatory or health agencies/organizations, e.g. IARC.

2- I suggest broadening the discrimination between the geogenic origin of Hg and its use and consequent redistribution due to anthropogenic activity, perhaps by replacing citation 2 (line 44) or adding new citations. It would also enrich the introduction if the regional and global impact of the problem addressed were highlighted.

3- I suggest reorganizing what is included between lines 48 and 51, replacing citation 6 with a more current one, clarifying the impact of the different states that the metal can have, the predominant forms in the environments of interest and the relative toxicity of each form.

4- Line 59, replace ‘toxicant’ by ‘toxic metal’ or ‘pollutant’.

5- Line 61 mentions the ability of bacteria to convert Hg into ‘non-toxic’ forms, clarify which would be the starting (toxic) forms and those derived from microbiological transformation. Perhaps this should be placed on line 66, after quote 18, where these forms are mentioned without the clarification of their toxicity.

6- Line 63, reference is made to the *mer* operon as one of the ‘most intriguing’ aspects of resistance to this metal, however its components, mode of action and relationship of the bacterial isolate selected (*P. stutzeri*) with this operon (if there are gene components previously found/described in this isolate, etc.) are not clearly detailed.

7- Lines 72- 74, *P. stutzeri* is mentioned as one of the bacterial isolates previously used in Hg remediation studies, I suggest including the main results to be highlighted in these studies, allowing *a priori* to know the effectiveness of this isolate in the remediation of sites contaminated with this metal. In this point, quotes 25 and 26 should be included (adding concrete removal data), reorganize.

8- The objective of the work should be reformulated (unifying what is stated in lines 75-76, 81-82 and 93-94), detailing the context of Hg bioremediation and the novelty of the application of SMF. To state clearly how the present study intends to contribute to solve the problem of interest.

9- Lines 82-88 Rewrite reference to previous studies with SMF, highlighting the main results (numerical data, % removal, etc.). Line 85 mentions that SMF ‘can accelerate the treatment of water’, rewrite, mentioning removal/remediation and data. A study analyzing the role of SMF in wastewater is mentioned (citation 27), and previously no wastewater was detailed as a source of Hg contamination.

At this point in the introduction, it is not clear which Hg contaminated matrix is of interest to the present work.

10- Lines 89-91 The fact that there are previous studies combining bacterial isolation (*P. stutzeri*) with SMF for the treatment of contaminated sources (citation 25) detracts from the originality of the work, despite the differential nature - organic vs. inorganic - of the pollutant in question.

11- Line 92 I suggest changing the term ‘decontamination’ to ‘remediation’.

**Materials and methods**

1- Lines 97-101, give more details about the area sampled, why it was selected, land use history, coordinates, number of samples taken, extent of area sampled.

2- Lines 102-104, detail which properties (physical and chemical) were determined and provide data of the correspondent equipment used.

3- Line 106, detail which heavy metals and major elements are referred to, range of detection/quantification, units of expression of the results.

4- Line 111, clarify whether the analyzer used (DMA-80) measured total mercury or allows the speciation of this to be quantified, brand of equipment.

5- Lines 114-118, up to this point it is not clear why they decided to add Hg instead of working with the reference concentrations in the sampled area, or if they have not quantified the metal in these samples. It is not clear at what concentrations of the metal they want to work with (ppm, M, Mm), by adding 148 mg HgSO_4_ to 100 ml H2Od you have prepared a 5 mM solution of the metal (or of the salt, whose molecular weight is predominantly contributed by mercury), you should clarify what are the desired working concentrations, why they have been selected and how they were prepared. In line 117, it is not understood what is meant by ‘at 50-gram dose’, as it cannot be equivalent to the concentration of Hg present in 1 kg of soil when adding that solution (as understood in the text). I suggest revising and rewrite providing all the necessary data for the understanding of the selected methodology. I suggest modifying the title of this subsection, I do not consider the term ‘ground doping’ to be appropriate.

6- Lines 119-125, If the bacterial isolation was obtained by working group members, which I deduce from the reference provided, this should be clarified. Which sequences are available in GenBank, 16S?, complete genome sequence?, provide the corresponding accession/bioproject numbers. If there is a history of the presence of genes involved in Hg tolerance/resistance in this isolate add it beforehand in the *Introduction* section.

7- Lines 127-141, in the bioaugmentation assays the authors should explain why they do not use the liquid inoculum of the bacteria (or sterile medium in the corresponding treatments) and do the washes mentioned in lines 127 and 128. In this respect, the composition of the media used (g/L), incubation/growth conditions of the bacteria, CFU/ml of the culture that proceeded to centrifugation should be included. This centrifugation was in duplicate? what is the volume of growth media centrifuged?, what was the total bacterial count used?, 7x10^10^ CFU/ml, 1.75x10^9^ CFU/ml?. In line 130 add the abbreviation ‘rpm’.

8- Line 132: **20 mg** of soil in 40 ml of medium (with or without bacteria) corresponds mainly to a test in liquid medium (the volume of soil used is almost negligible), which considerably biases the conclusions that can be drawn for this matrix, on which the results are presented in the *Abstract* and throughout the work.

9- From what is written in lines 134 to 136 I interpret that there are 3 different treatments (in flask 1, flask 2, flasks 3+4), in this description the sterile soil without bioaugmentation is not included as a control, which I suggest adding. So far it is interpreted that the n of treatment replicates is 1 (for the first two treatments) and 2 for the non-sterile soil without bioaugmentation (flasks 3 and 4), which makes it very weak statistically. Whether what is stated in line 139 corresponds to replicates of these treatments should be clarified above, or whether it refers to temporal replicates of the trials. It is also not clarified that the soil sample used corresponds to the soil added with Hg, clarify, explain in detail all the procedures so that those who did not carry out the work can clearly understand the methodological steps followed.

10- Lines 138-139 ‘Bacterial counts and mercury analysis were conducted on a weekly basis’, detail here the methodology used for this purpose, did they quantify total Hg, speciation? If these parameters were measured weekly for a total period of 28 days there should be 4 sampling and data collection times (does not correspond with the times shown in Fig. 1-4), please clarify.

11- Lines 140-141, to which aseptic conditions do you refer? in which procedure were they used? through which materials or equipment were they achieved?

12- Lines 142-152, describe the equipment used to expose the samples to MFS, I suggest attaching a picture of it.

13- Lines 152-153 ‘prior to the commencement of the observation period’, refers to the soil samples prior to disposition in the previous bioaugmentation test, it is not easily detectable if it was the same test (as interpreted in the Abstract) or not, needs to be clarified, reformulated, at what time the soil samples were subjected to this field, pre or post dilution in the 40 ml of medium, I suggest to attach a picture of it.

14- Lines 156-162, why are treatments described again, can this section not be summarily unified with the one previously describing the different treatments in the Flasks, why are they now ‘Bottles’ 1-4, when I understand that they refer to the treatments already described.

**Results**

1- I suggest presenting the different results in accordance with what is described in the *Materials and Methods* section, without the divisions presented in the subtitles of lines 202-203, 224-225, 246.

2- Line 223 mentions a treatment that was not described in the *Materials and Methods* section, ‘non-bioaugmented sterile soil’.

**Discussion**

1- Line 264, I suggest replacing the term ‘decontamination’ with remediation.

2- Do you consider SMF as an ‘economically viable’ methodology? in which cases? for which matrices? would it be possible to use it on a large scale? discuss these, pros and cons of this methodology, potential impact on native microbiota. It remains to add to this section previous work carried out to remediate Hg, its main results, and contrasts with what is observed in the current work. A considerable part of the discussion refers to other contaminants, which are not the one of interest, nor is there any discussion on the mechanisms that allow *P. stutzeri* LBR to remediate the metal.
