# Supplementary material for: Novel method for combining microbial bioremediation with static magnetic fields to remediate mercury-contaminated soils
Source: PLoS One. 2025 Aug 22;20(8):e0330872. doi: 10.1371/journal.pone.0330872 (PMC12373231; doi:10.1371/journal.pone.0330872)
Supplement: Attachment — Submitted filename: Response to reviewers.docx [file pone.0330872.s007.docx]

**14 /06/ 2025**	Laboratory of Biochemistry and Molecular Biology

Faculty of Sciences of Bizerte

7021, Zarzouna, Bizerte

TUNISIA

**Response to reviewer**

**Dear Editor-in-Chief of PLOS ONE,**

Thank you very much for your interest,

Authors are also thankful for reviewers for their interest and precious comments,

Please find below the answers to reviewer comments:

**Note:**

Line order was changed between the old manuscript and the revised manuscript.

In response to the reviewers’ comments, we have revised the manuscript accordingly.

The changes made following Reviewer 1’s suggestions are highlighted in yellow, and those addressing Reviewer 2’s feedback are highlighted in blue.

Review Comments to the Authors

**Reviewer #1:**

**Abstract**

1. I suggest revising the spelling and formatting, respecting the spacing throughout the text.
We thank reviewer 1 for this comment, the manuscript was re-read by the Proof-Reading Service (please view the certificate).

2. Do not use abbreviations, particularly in the case of bacterial isolate nomenclature.

Thank you for your comment. I have removed all abbreviations from the abstract, particularly those related to bacterial isolate nomenclature, to improve clarity.

3. When starting to refer to the results obtained, emphasize the observed effect (increase, decrease, etc.) on Hg removal instead of ‘The results demonstrated the impact of SMF on...’.
We thank the reviewer 1 for this comment; the sentence " The results demonstrated the impact of SMF on..." is replaced by the sentence " Mercury remediation was enhanced by SMF ……." at line 32 of the abstract.

4. The statement that the combination of SMF and P. stutzeri ‘accelerated’ metal remediation in sterile soil, compared to SMF alone, suggests that the effect was observed in a shorter treatment time. If this was the case, I suggest clarification, otherwise ‘accelerated’ should be replaced by increased/promoted removal by x %, relative to SMF alone.

We thank Reviewer 1 for this comment. In the revised manuscript, we have replaced the word ‘accelerated’ with ‘increased’ (as suggested) on line 34	.

**Introduction :**

1- Line 42, where it is argued that mercury is a bioaccumulative ‘toxin’, I suggest revision of the term. Hg can be classified as a ‘toxic’ metal, but not a toxin, which presupposes a biological origin of the compound in question. The above-mentioned citation of reference [1] does not claim that it is a toxin. I suggest adding its classification according to regulatory or health agencies/organizations, e.g. IARC.

We thank Reviewer 1 for this comment. In the revised manuscript, we have replaced the word ‘toxin’ with ‘toxic’ (as suggested) on line 45. We have included the classification of mercury in accordance with the guidelines provided by IARC and UNEP at line 49.

2- I suggest broadening the discrimination between the geogenic origin of Hg and its use and consequent redistribution due to anthropogenic activity, perhaps by replacing citation 2 (line 44) or adding new citations. It would also enrich the introduction if the regional and global impact of the problem addressed were highlighted.

Thank you for your valuable feedback. I would like to confirm that I have carefully addressed your comment regarding the sources and impacts of mercury. As suggested, I have included references to both natural sources (e.g., volcanic eruptions and weathering of rocks) and anthropogenic activities (e.g., coal combustion and mining) based on the works of Sonke et al. (2023). Additionally, I have emphasized the global dispersion and bioaccumulation of mercury, highlighting its environmental and health risks, as supported by Outridge et al. (2018) and UNEP (2018). The corrections have been made in the manuscript between line 50 and line 53.

3- I suggest reorganizing what is included between lines 48 and 51, replacing citation 6 with a more current one, clarifying the impact of the different states that the metal can have, the predominant forms in the environments of interest and the relative toxicity of each form.

Thank you for your insightful suggestion. I have carefully reorganized the content between lines 48 and 51 as recommended. Additionally, I have replaced citation 6 with more current and relevant Kumar et al. (2024). To address your comment, I have clarified the impact of the different states that mercury can have, specified the predominant forms in the environments of interest, and discussed the relative toxicity of each form. These changes aim to provide a clearer and more up-to-date discussion of the topic. The corrections have been made in the manuscript between line 54 and line 69.

4- Line 59, replace ‘toxicant’ by ‘toxic metal’ or ‘pollutant’.

We thank Reviewer for this comment. Following the revisions in the text, this word has been removed.

5- Line 61 mentions the ability of bacteria to convert Hg into ‘non-toxic’ forms, clarify which would be the starting (toxic) forms and those derived from microbiological transformation. Perhaps this should be placed on line 66, after quote 18, where these forms are mentioned without the clarification of their toxicity.

Thank you for your valuable comment. we have carefully clarified the toxic forms of mercury (such as methylmercury and inorganic Hg²⁺) and the non-toxic or less toxic forms resulting from microbiological transformation (e.g., elemental mercury Hg⁰). As you recommended, we have also repositioned this clarification to line 66, after citation 18, to ensure a more logical and coherent presentation of the information. The corrections have been made in the manuscript between line 74 and line 85.

6- Line 63, reference is made to the mer operon as one of the ‘most intriguing’ aspects of resistance to this metal, however its components, mode of action and relationship of the bacterial isolate selected (P. stutzeri) with this operon (if there are gene components previously found/described in this isolate, etc.) are not clearly detailed.

We thank the reviewer for this relevant suggestion. We have expanded the section on the *mer* operon by detailing its components, mode of action, and its connection with *Pseudomonas stutzeri*. We have added an explanation of the merA, merR, merT, merP, and merB genes, as well as their role in mercury detoxification. Additionally, we have mentioned that *P. stutzeri* possesses these genes, which grants it a demonstrated ability to resist and detoxify mercury. The corrections have been made in the manuscript between line 91 and line 96.

7- Lines 72- 74, P. stutzeri is mentioned as one of the bacterial isolates previously used in Hg remediation studies, I suggest including the main results to be highlighted in these studies, allowing a priori to know the effectiveness of this isolate in the remediation of sites contaminated with this metal.

Thank you for your comment. In this study, we present the first investigation of mercury degradation by the *P. stutzeri* LBR strain selected in our laboratory. In contrast, the Zheng et al. (2017) demonstrated that the marine strain *P. stutzeri 273* can resist mercury concentrations up to 50 µM and eliminate up to 94% of mercury in culture. However, this degradation rate was measured in an artificial LB medium, not under real environmental conditions such as marine sediments or contaminated seawater. The corrections have been made in the manuscript between line 94 and line 101.

In this point, quotes 25 and 26 should be included (adding concrete removal data), reorganize.

Thank you for your comment. We have included quotes 25 and 26, added the concrete removal data, and reorganized the section as suggested. “Specifically, it was capable of degrading 87% of 1,1,1-trichloro 2,2-bis(p-chlorophe´nyl) ethane (DDT) and 83% of Benzo(a)pyrene (BaP) in the environment [25]. Moreover, when bioaugmented individually in non-sterile soil, it effectively reduced Pb concentrations by 58.07% [26]. Following the modifications made to the manuscript, the line numbers and references have changed. The corrections have been made in the manuscript between line 103 and line 106.

8- The objective of the work should be reformulated (unifying what is stated in lines 75-76, 81-82 and 93-94), detailing the context of Hg bioremediation and the novelty of the application of SMF. To state clearly how the present study intends to contribute to solve the problem of interest.

Thank you for your comment. As recommended, I have reworded the study objective between lines 116 and 124 in the revised manuscript.

9- Lines 82-88 Rewrite reference to previous studies with SMF, highlighting the main results (numerical data, % removal, etc.).

Line 85 mentions that SMF ‘can accelerate the treatment of water’, rewrite, mentioning removal/remediation and data. A study analyzing the role of SMF in wastewater is mentioned (citation 27), and previously no wastewater was detailed as a source of Hg contamination. At this point in the introduction, it is not clear which Hg contaminated matrix is of interest to the present work.

We thank the reviewer for this valuable comment. Since the primary focus of this study is polluted soil, we have clarified this point at the beginning of the paragraph (line 108) before discussing previous studies that applied SMF for the bioremediation of soils and wastewater.

We have changed [can accelerate the treatment of water] to [enhanced wastewater remediation] (Line 112).

We have revised this section to include quantitative results from previous SMF studies (Line 109 to 115)

10- Lines 89-91 The fact that there are previous studies combining bacterial isolation (P. stutzeri) with SMF for the treatment of contaminated sources (citation 25) detracts from the originality of the work, despite the differential nature - organic vs. inorganic - of the pollutant in question.

Citation 25 (corresponds to citation 28 in the revised manuscript) refers to the work of Mansouri et al. (2019), conducted in our laboratory. The difference between that study and the current work is not limited to the nature of the pollutants investigated (organic vs. inorganic), but lies more importantly in our aim to understand how a static magnetic field (SMF) can influence distinct metabolic pathways involved in bioremediation. Indeed, it is well known that bacterial remediation mechanisms differ depending on the type of pollutant: in the case of an organic pollutant, bacteria can use it as a carbon source, whereas for a heavy metal, they tend to accumulate, transform, or precipitate it. Furthermore, bioremediation mechanisms can vary significantly not only from one heavy metal to another, but also between different bacterial species. Moreover, very limited data are currently available on this subject, which we consider to be highly promising. The mechanisms by which SMF influences bioremediation remain poorly understood, and we believe it is essential to further investigate how a static magnetic field can accelerate bioremediation. Which pathways are involved? How is it possible to enhance bioremediation using two different types of pollutants (organic and inorganic), despite the fact that the metabolic mechanisms involved are fundamentally different?

In conclusion, based on all these arguments, we believe that this work is original and fits well within the scope of this project, which our team considers of high interest, as it contributes to a better understanding of the mechanisms of SMF action in bioremediation, with potential applications in the future.

11- Line 92 I suggest changing the term ‘decontamination’ to ‘remediation’.

We thank the reviewer for this relevant suggestion. In the revised manuscript, we have replaced the word ‘decontamination ’ with ‘remediation’ (as suggested) on line 119.

**Materials and Methods**

1- Lines 97-101, give more details about the area sampled, why it was selected, land use history, coordinates, number of samples taken, extent of area sampled.

Thank you for your valuable comment. We have revised Lines 97-101 to provide more details about the sampled area as requested. The soil samples were collected in the Kasserine region, located in the west-central part of Tunisia, within an industrial zone (GPS: 35.168534, 8.817806). This site was chosen due to its historical industrial activity for the purpose of industrial waste disposal for more than two decades. A total of 30 soil samples were collected over an area of approximately 300 m² to ensure a representative analysis of the site. These additional details have been incorporated into the revised manuscript to improve clarity and context (Line 128 to 132).

2- Lines 102-104, detail which properties (physical and chemical) were determined and provide data of the correspondent equipment used.

Thank you for your comment. The requested details (physical/chemical properties and corresponding equipment specifications) have been added in Lines 148 to 161 of the revised manuscript.

3- Line 106, detail which heavy metals and major elements are referred to, range of detection/quantification, units of expression of the results.

We thank the reviewer for this valuable comment. In response, we have detailed the heavy metals and major elements referred to in the study (Line 162 to 163), including the units used to express the results (mg/kg dry matter) Line 167. Table 1 below presents the detection and quantification limits for the analyzed elements by (ICP-OES) This table will be included in the Supporting Information section of the article. The LOD and LOQ values for mercury measured by the DMA-80 are included at line 170 to 171 of the revised manuscript.

4- Line 111, clarify whether the analyzer used (DMA-80) measured total mercury or allows the speciation of this to be quantified, brand of equipment.*

We thank the reviewer for this comment. We confirm that the DMA-80 analyzer measures total mercury, and the brand of the equipment is Milestone. This clarification has been added to the manuscript for accuracy (Line 168 to 171).

5- Lines 114-118, up to this point it is not clear why they decided to add Hg instead of working with the reference concentrations in the sampled area, or if they have not quantified the metal in these samples. It is not clear at what concentrations of the metal they want to work with (ppm, M, Mm), by adding 148 mg HgSO4 to 100 ml H2Od you have prepared a 5 mM solution of the metal (or of the salt, whose molecular weight is predominantly contributed by mercury), you should clarify what are the desired working concentrations, why they have been selected and how they were prepared. In line 117, it is not understood what is meant by ‘at 50-gram dose’, as it cannot be equivalent to the concentration of Hg present in 1 kg of soil when adding that solution (as understood in the text). I suggest revising and rewrite providing all the necessary data for the understanding of the selected methodology. I suggest modifying the title of this subsection, I do not consider the term ‘ground doping’ to be appropriate.

We thank the reviewer for this comment. After analyzing the mercury content in the soil from the sampling area (soil S1), we detected a very low mercury concentration of 0.3 mg/kg DM (see Table 1 in the revised manuscript). In fact, natural mercury concentrations in uncontaminated soils typically range from 0.003 and 0.5 mg/kg DM Arbestain et al. (2OO9). In residential soils, intervention thresholds, although they vary across national regulations, generally converge around values between 7 and 10 mg/kg DM (Bell 2016 , UNEP 2013). These findings indicate that soil S1 was not contaminated with mercury. Therefore, we artificially added mercury to achieve adequate concentrations for bioremediation. The explanations have been made in the manuscript : (Line 136 to 137) and (Line 249 to 258).

Additionally, as you suggested, we have changed the section title from “Ground doping” to “Soil sampling and preparation” (Line 127).

6- Lines 119-125, If the bacterial isolation was obtained by working group members, which I deduce from the reference provided, this should be clarified. Which sequences are available in GenBank, 16S?, complete genome sequence?, provide the corresponding accession/bioproject numbers. If there is a history of the presence of genes involved in Hg tolerance/resistance in this isolate add it beforehand in the Introduction section.

We thank the reviewer for this important comment. We confirm that the bacterial isolation was performed by members of our working group, as indicated in the reference provided. The sequences available in GenBank are 16S rRNA, and the corresponding accession number is KC157911. Regarding the history of genes involved in Hg tolerance/resistance, it is well-documented in the literature that *Pseudomonas stutzeri* strains often carry the *mer* operon, which confers resistance to heavy metals. However, for our specific isolate *Pseudomonas stutzeri* LBR, the detection of the *mer* operon has not been performed, and further studies would be needed to explore this aspect.

7- Lines 127-141, in the bioaugmentation assays the authors should explain why they do not use the liquid inoculum of the bacteria (or sterile medium in the corresponding treatments) and do the washes mentioned in lines 127 and 128.

We thank the reviewer for this question.To ensure rapid growth, the overnight pre-culture was performed in a nutritionally rich liquid medium (LB medium). For this reason, washing steps are critical. This information has been added to the revised manuscript version (Line 190).

In this respect, the composition of the media used (g/L), incubation/growth conditions of the bacteria, CFU/ml of the culture that proceeded to centrifugation should be included. This centrifugation was in duplicate? what is the volume of growth media centrifuged?, what was the total bacterial count used?, 7x1010 CFU/ml, 1.75x109 CFU/ml?.

We thank the reviewer for their comments. In the following paragraph, which we have added to the revised manuscript, we have included all the requested information:

[We prepared four independent overnight precultures of *P. stutzeri* LBR in 10 mL of LB medium at 30 °C, with shaking at 100 rpm. The following morning, the bacterial pellets were washed three times with M9 medium. Each wash was followed by centrifugation at 5,000 rpm for 5 minutes at 4 °C. The final pellets were resuspended in 50 mL of sterile M9 medium to reach an optical density at 600 nm (OD600) of 0.1, corresponding to approximately 7x10^9^ cells. These four bacterial suspensions were subsequently used to inoculate the four flasks prepared for the bioaugmentation experiment.], Line 190 to 194.

In line 130 add the abbreviation ‘rpm’.

We have added the abbreviation ‘rpm’ Line 192.

8- Line 132: 20 mg of soil in 40 ml of medium (with or without bacteria) corresponds mainly to a test in liquid medium (the volume of soil used is almost negligible), which considerably biases the conclusions that can be drawn for this matrix, on which the results are presented in the Abstract and throughout the work.

We thank the reviewer for this observation. The reviewer is correct—this was a typographical error. The correct value is **20 g** of soil in 40 ml of medium, not 20 mg. This has been corrected in the manuscript on line 195.

9- From what is written in lines 134 to 136 I interpret that there are 3 different treatments (in flask 1, flask 2, flasks 3+4), in this description the sterile soil without bioaugmentation is not included as a control, which I suggest adding. So far it is interpreted that the n of treatment replicates is 1 (for the first two treatments) and 2 for the non-sterile soil without bioaugmentation (flasks 3 and 4), which makes it very weak statistically. Whether what is stated in line 139 corresponds to replicates of these treatments should be clarified above, or whether it refers to temporal replicates of the trials. It is also not clarified that the soil sample used corresponds to the soil added with Hg, clarify, explain in detail all the procedures so that those who did not carry out the work can clearly understand the methodological steps followed.

We thank the reviewer for this comment. Indeed, we have four different treatments. Flask 1: Non-sterile soil supplemented with Hg, M9 (pyruvate), and P. stutzeri LBR. Flask 2: Sterile soil supplemented with Hg, M9 (pyruvate), and P. stutzeri LBR. Flask 3: Non-sterile soil supplemented with Hg and M9 (pyruvate). Flask 4: Sterile soil supplemented with Hg and M9 (pyruvate).

In all treatments, we used soil supplemented with Hg. To ensure reproducibility, three temporal replicates were conducted for each treatment, meaning that the entire experiment was repeated three times under identical conditions. We have incorporated all these suggestions into the revised manuscript on line 200 to 208.

10- Lines 138-139 ‘Bacterial counts and mercury analysis were conducted on a weekly basis’, detail here the methodology used for this purpose, did they quantify total Hg, speciation? If these parameters were measured weekly for a total period of 28 days there should be 4 sampling and data collection times (does not correspond with the times shown in Fig. 1-4), please clarify.

We thank the reviewer for this observation. The experiment lasted 30 days, and sampling was conducted every 15 days (not weekly). This has been clarified in the revised manuscript on line 204.

11- Lines 140-141, to which aseptic conditions do you refer? in which procedure were they used? through which materials or equipment were they achieved?

We thank the reviewer for this comment. The aseptic conditions refer to the use of sterilized materials (e.g., autoclaved glassware, sterile pipettes, and gloves) and procedures performed under a laminar flow hood to prevent contamination during bacterial counts and mercury analyses. These details have been added to the revised manuscript from line 208 to line 210 for clarity.

12- Lines 142-152, describe the equipment used to expose the samples to SMF, I suggest attaching a picture of it.

I have added the static magnetic field picture to the Supporting Information. Please consult the Supporting Information section located after the references.

13- Lines 152-153 ‘prior to the commencement of the observation period’, refers to the soil samples prior to disposition in the previous bioaugmentation test, it is not easily detectable if it was the same test (as interpreted in the Abstract) or not, needs to be clarified, reformulated, at what time the soil samples were subjected to this field, pre or post dilution in the 40 ml of medium, I suggest to attach a picture of it.

We thank the reviewer for this comment. Soil samples were subjected to this process (SMF) after dilution in 40 ml of medium. After preparing the four treatments, the content of the flasks (soil supplemented with Hg, M9 (pyruvate) with or without *P. stutzeri* LBR) was then transferred into a double-walled glass vial and subjected to a magnetic field of 260 mT for a period of 12 hours. Subsequently, the four flasks were placed in a shaking water bath at 30°C for 30 days to monitor Hg concentration and bacterial enumeration. These details have been added to the revised manuscript from line 223 to line 227 for clarity.

I have added the static magnetic field picture to the Supporting Information. Please consult the Supporting Information section located after the references.

14- Lines 156-162, why are treatments described again, can this section not be summarily unified with the one previously describing the different treatments in the Flasks, why are they now ‘Bottles’ 1-4, when I understand that they refer to the treatments already described.

We thank the reviewer for this observation. The reviewer is absolutely correct—this paragraph was an unnecessary repetition of the treatments already described. We have deleted this section entirely to avoid redundancy and improve the flow of the manuscript.

**Results**

1-I suggest presenting the different results in accordance with what is described in the Materials and Methods section, without the divisions presented in the subtitles of lines 202-203, 224-225, 246.

I thank the reviewer for this suggestion, which I have taken into account in the revised version of the manuscript.

2- Line 223 mentions a treatment that was not described in the Materials and Methods section, ‘non-bioaugmented sterile soil’.

We thank the reviewer for this observation. The treatment mentioned as "non-bioaugmented sterile soil" corresponds to Flask 4, which has now been explicitly described in the Materials and Methods section for clarity (Line 202).

**Discussion**

1- Line 264, I suggest replacing the term ‘decontamination’ with remediation.

We thank Reviewer for this comment. In the revised manuscript, we have replaced the word ‘decontamination’ with ‘remediation’ (as suggested) on line 325.

2- Do you consider SMF as an ‘economically viable’ methodology? in which cases? for which matrices? would it be possible to use it on a large scale? discuss these, pros and cons of this methodology, potential impact on native microbiota. It remains to add to this section previous work carried out to remediate Hg, its main results, and contrasts with what is observed in the current work. A considerable part of the discussion refers to other contaminants, which are not the one of interest, nor is there any discussion on the mechanisms that allow P. stutzeri LBR to remediate the metal.

Thank you for your valuable suggestions. The discussion section has been fully revised to address all the points raised. We have considered the economic viability and scalability of the SMF approach, its applicability to various matrices, and its impact on native microbiota. We also added recent references on mercury remediation, highlighted key results from previous studies, and compared them with our findings. The discussion now focuses exclusively on mercury and includes a section on the mechanisms involved in *P. stutzeri* LBR-mediated remediation (Line 382 to 455).

**Reviewer #2:**

1. Consider reorganizing the content to progress from general mercury contamination issues to the specific focus of your study. For example:
   - Mercury as an environmental pollutant (current impacts and sources).
   - Current remediation challenges (limitations of traditional methods).
   - Potential of bioremediation (your approach and objectives).

We sincerely thank the reviewer for this insightful suggestion. We have thoroughly revised the introduction to follow the recommended progression: starting with general issues related to mercury contamination, followed by the limitations of current remediation methods, and concluding with the potential of bioremediation and the specific objectives of our study. This restructuring aims to improve the clarity and logical flow of the manuscript.

2. Some sentences are lengthy and contain multiple ideas. Consider breaking them down to improve clarity.

We thank reviewer 2 for this comment, the manuscript was re-read by the Proof-Reading Service (please view the certificate).

3. For example: You mention that *"a variety of bacteria have the capacity to resist toxic forms of mercury and subsequently convert them into non-toxic forms."*
   - It would be helpful to briefly explain the mechanisms involved in these conversions.

We thank the reviewer for this helpful suggestion. As requested, we have added a brief explanation of the microbial mechanisms involved in mercury conversion. This includes the role of the *mer* operon, particularly the enzymes MerA and MerB, in reducing Hg²⁺ to elemental mercury (Hg⁰) and breaking down organomercurials. These additions can be found in the revised manuscript from lines 54 to 85 and further elaborated between lines 398 and 400.

- You could also mention if P. stutzeri LBR has been compared with other strains for effectiveness.

We thank the reviewer for this valuable suggestion. A clarification has been added in the revised manuscript (lines 392 to 417) indicating that *Pseudomonas stutzeri* LBR has been compared to other strains in terms of mercury resistance and bioremediation efficiency.

4. The study objective is stated clearly at the end, but consider emphasizing what practical applications or next steps could follow from this research.

The response to this comment has been incorporated into the manuscript, specifically between lines 442 and 455. We have strengthened the final section of the discussion to better emphasize the potential practical applications of this research as well as the prospects for future work.

5. The material and method section is well-structured, but it could benefit from clearer subheadings and a more logical flow. Consider structuring it as follows for better readability: Soil Sampling and Preparation/ Soil Analysis (Physical, Chemical, and Heavy Metal Concentration)/ Bacterial Strain and Inoculation/ Bioaugmentation Tests/ Static Magnetic Field (SMF) Application/ Monitoring and Analysis/ Statistical Analysis.

We thank Reviewer for this comment. We have revised the Materials and Methods section as suggested, with clearer subheadings and a more logical flow to improve readability.

6. Specify if the mercury solution was mixed uniformly to ensure even distribution in the soil.

We thank Reviewer for this comment. The following sentence has been added to the revised manuscript to clarify this point at line 145.

- Clarify why pH 7 was chosen and its significance in mercury solubility/stability.

We sincerely thank the reviewer for their valuable comment regarding the pH selection. The neutral pH (7) was chosen to: maintain Hg²⁺ solubility and prevent precipitation (Morel et al. (1998)) (Line 139)

- Indicate whether control groups were included for comparative purposes.

We thank Reviewer for this comment. Yes, we included control groups in our study. These consisted of soil samples supplemented with mercury (Hg), which were either sterilized or non-sterilized, and not subjected to bioaugmentation by *P. stutzeri* LBR. These control groups were used for comparative purposes to assess the specific effects of bioaugmentation and sterilization on mercury behavior in the soil.

- The bacterial inoculum concentration (70 x 10⁹ cells) should mention if it was determined through OD measurements or CFU counting for better reproducibility.

We thank the reviewer for raising this important methodological point. We confirm that: The bacterial concentration was determined by OD600 measurement based on a pre-established standard curve correlating OD600 with CFU counts for strain *Pseudomonas stutzeri* LBR (Line 194).

We have corrected a typographical error in the concentration value: the correct inoculum concentration is 7 × 10⁹ cells/mL (not 70 × 10⁹ cells/mL as previously stated). This correction has been made throughout the manuscript (lines 194).

- Mention if post-hoc tests were applied to detect significant differences between groups.

We thank Reviewer for this comment. Post-hoc tests (Tukey test) were applied to detect significant differences between groups, as introduced in the revised text (line 232).

- Instead of "significance level of p < 0.05," consider rephrasing to "statistical significance was set at p < 0.05."

We thank Reviewer for this comment. In the revised manuscript, we have replaced the sentence “significance level of p < 0.05” with “statistical significance was set at p < 0.05.” (as suggested) on line 233.

7. Ensure correct citation formatting, e.g.,
   - "[4 , 5]" should be "[4,5]" (remove spaces before commas).

Thank you for your comment. We have carefully reviewed the manuscript and removed all spaces before commas as requested. The text has been updated accordingly.

- Proper reference order for standards like "AFNORNF ISO 11265" should follow a uniform style.

We thank Reviewer for this comment. All standards cited in the manuscript have been revised to follow the uniform ISO referencing style, in accordance with international norms.

- Some paragraphs contain excessive detail; simplifying them can make the section more reader-friendly.

We thank Reviewer for this comment. We have carefully reviewed the section and simplified paragraphs containing excessive detail to improve readability and make the content more reader-friendly (Proofreading certificate).

- Increase the clarity of tables and charts.

Thank you for your valuable feedback. We have revised the tables and charts to improve their clarity. Specifically, we have adjusted the formatting, labels, and legends to ensure they are more readable and informative. These changes are reflected in the updated manuscript.

8. Summarize the key findings more clearly, highlighting the effectiveness of bioaugmentation and SMF's impact.

We thank the reviewer for this suggestion. We have now clearly summarized the key findings regarding bioaugmentation effectiveness and SMF impact in the revised manuscript (line 330 to 350).

9. When presenting comparative results, such as the 49.36% and 72.49% remediation rates, consider briefly explaining why sterile soils showed a higher improvement compared to non-sterile soils. This helps provide context for the reader.
10. Clarify mechanisms where necessary; for instance, when discussing microbial competition in non-sterile soils, elaborate briefly on how this impacts mercury uptake.

Answers to Questions 9 and 10: We sincerely thank the reviewer for these insightful questions regarding (1) the enhanced bioremediation efficiency in sterile soils and (2) the specific mechanisms of microbial competition affecting mercury uptake (We have carefully addressed both points in the revised manuscript through the addition of an explanatory paragraph (lines 356 to 370 in the Discussion section).

Instead of saying "published studies," specify which studies to strengthen credibility.

Thank you for your valuable comment. We have replaced “Published studies” with “Several studies, such as those by Niu et al. (2014) and Zaidi et al. (2014)”. This modification has been incorporated into the revised manuscript (lines 421 to 422)

11. A brief mention of potential future experiments to confirm these mechanisms could enhance the scientific rigor of the document.
12. Expand on future research opportunities, such as optimizing SMF parameters (intensity, duration), exploring its application in real-world contaminated sites, and assessing long-term environmental impacts.

Answers to Questions 11 and 12: We thank the reviewer for these two suggestions. A new paragraph has been added in the revised manuscript from line 466 to 455.

13. Ensure uniform formatting in terms of punctuation, spaces, and abbreviations.

Thank you for your valuable comment. We have carefully reviewed the manuscript to ensure uniform formatting in terms of punctuation, spaces, and abbreviations. All necessary corrections have been made to maintain consistency throughout the text (Proofreading certificate).

14. For example, journal names should follow a consistent format (e.g., *Atmos. Environ.* or *Atmospheric Environment*, but not a mix of both).

- Instead of writing the DOI as a URL, it's better to present it in the standard format: doi:10.xxxx/xxxx.
- Example:
  https://doi.org/10.1016/S1352-2310(97)00293-8 → doi:10.1016/S1352-2310(97)00293-8
- Ensure a consistent style for author initials (e.g., "W.H. Schroeder" vs. "WH Schroeder").
- Remove unnecessary spaces between initials.
- Ensure all references have complete citation details, such as volume, issue, page numbers, and publication year.

We sincerely thank the reviewer for their insightful comments, all of which we have carefully addressed in the revised manuscript. Each suggestion has been thoroughly considered and incorporated where appropriate to improve the quality and clarity of our work.

Waiting for your reply,

My thanks in advance,

Dr Chiraz ABBES
