# Supplementary material for: Novel method for combining microbial bioremediation with static magnetic fields to remediate mercury-contaminated soils
Source: PLoS One. 2025 Aug 22;20(8):e0330872. doi: 10.1371/journal.pone.0330872 (PMC12373231; doi:10.1371/journal.pone.0330872)
Supplement: Attachment — Submitted filename: Response to Reviewer.docx [file pone.0330872.s008.docx]

Laboratory of Biochemistry and Molecular Biology	03/08/2025

Faculty of Sciences of Bizerte

7021, Zarzouna, Bizerte

TUNISIA

**Response to reviewer**

**Dear Editor-in-Chief of PLOS ONE,**

Thank you very much for your interest.
The authors would also like to thank the reviewer for their interest and valuable comments.
Below are our responses to Reviewer 3’s comments:

**Reviewer 3**

**Technical quality**

1. Inconsistent Results Reporting (Page 2, Lines 33–39; Page 10–12)
The Abstract states SMF accelerated remediation by 49.36% (non-sterile) and 72.49% (sterile). In contrast, Results sections report 81.77% (non-sterile) and 87.17% (sterile) reductions in Hg concentration.
Action: Reconcile these discrepancies. Define how "acceleration" is calculated (e.g., percentage increase in remediation rate vs. control). Provide a unified metric throughout.

Thank you for your comment. To better explain the percentages obtained, you will find below a summary table of the key results.

|  |  | Mercury reduction rate (%) | | Percentage increase mercury in bioremediation efficiency (%) | |
| --- | --- | --- | --- | --- | --- |
|  |  | Without SMF | With SMF | Without SMF | With SMF |
| Non-sterile soil | Non- bioaugmented | 32.11 | 32.42 | 55.96 - 32.11=  23.85 | 81.77- 32.42=  49.36 |
|  | bioaugmented | 55.96 | 81.77 |  |  |
| Sterile soil | Non-bioaugmented | 7.53 | 14.68 | 45.63 -7.53 =  38.1 | 87.17- 14.68=  72.49 |
|  | bioaugmented | 45.63 | 87.17 |  |  |

The SMF enhances the efficiency of bioremediation in both types of soil. In non-sterile soil, in the absence of SMF, bioaugmentation results in only a 23.85% improvement, compared to 49.36% in the presence of SMF. Similarly, in sterile soil, the improvement increases from 38.1% without SMF to 72.49% with SMF. Thus, SMF acts as an amplifier of bioremediation efficiency, particularly in sterile soil. However, in the absence of bioaugmentation (introduced bacteria) or indigenous microbial flora, the effect of SMF remains limited. It can therefore be concluded that the synergy between SMF and bioaugmentation is essential to optimize and accelerate mercury bioremediation.

2. Unclear Experimental Controls (Page 7, Lines 133–141)
Flasks 3–4 are described as "non-sterile soil + M9" without specifying if P. stutzeri LBR was included. This ambiguity complicates interpretation of bioaugmentation effects.
Action: Explicitly label control groups (e.g., *"Flask 3: Non-sterile soil + M9 (no bacteria)"*).

**Thank you for your comment. We have added ‘without *P. stutzeri* LBR’ to the composition of flasks 3 and 4. Please see line 203 and 204.**

3. Page 12, Lines 242–245:

Issue: Attribution of bacterial decline to pyruvate depletion is speculative without supporting data (e.g., pyruvate measurements). Recommendation: Provide data on carbon source depletion or temper conclusions.

Thank you for the comment. We have tempered the conclusion accordingly by adding the sentence: "However, further analysis, including direct measurements of pyruvate levels, would be necessary to confirm this hypothesis." Ligne 381.

4. Unsupported Mechanistic Claims (Page 15, Lines 331–334)
The proposal that SMF enhances mercuric reductase (MerA) activity lacks experimental validation (e.g., enzyme assays).
Action: Include MerA activity data under SMF exposure OR reframe this as a testable hypothesis (e.g., "SMF may stimulate MerA, warranting further study").

We fully agree with the reviewer’s comment. However, we could not locate the statement regarding the effect of SMF on MerA activity in the indicated lines (331–334) or elsewhere in the manuscript.

**Language et clarity**

1. Throughout: o Issue: Inconsistent italicization of bacterial names (e.g., *Pseudomonas stutzeri* vs. Pseudomonas stutzeri). o Recommendation: Italicize all genus/species names (e.g., *P. stutzeri* LBR).

Thank you for your comment. The formatting of all genus and species names has been checked and corrected to ensure consistent italicization throughout the manuscript.

2. Page 3, Line 64:

Issue: "borne on plasmids" → should be "born on plasmids."

Correction: Replace with "born."

Thank you for the comment. The expression has been updated to "born on plasmids" as requested, and the correction has been made in line 80 of the revised manuscript.

3. Page 10, Lines 210–214:

Redundant phrasing ("non-bioaugmented non-sterile soil").

Correction: Simplify to "non-bioaugmented soil."

Thank you for the suggestion. The phrase has been revised to **"in non-sterile soil without bioaugmentation"** in the revised manuscript, Line 274.

4. Page 13, Lines 268–269: o Issue: Awkward syntax ("deployment of SMF in environmental engineering with the objective of..."). o Correction: "We deployed SMF in environmental engineering to enhance soil mercury remediation."

Thank you for your suggestion. We replaced the sentence with:
**"Our research focuses on deploying an SMF in environmental engineering to enhance soil mercury remediation." Line 331.**

**Methodology & Reporting**
1. Ambiguous Hg Dosing (Page 6, Line 118)
The protocol (148 mg HgSO₄ in 100 mL solution added to 1 kg soil) does not specify the final soil Hg concentration.
Action: State Hg concentration post-doping (e.g., "resulting in X mg Hg/kg soil").

Thank you for your comment. We have added the final mercury concentration (10.15 mg Hg/kg soil) to the protocol description line 145 as requested.

2. Background Metal Levels (Page 9, Table 1)
Elevated Fe (6504 mg/kg) and Cr (341.03 mg/kg) suggest potential soil contamination. Their impact on Hg remediation is unaddressed.
Action: Briefly discuss whether background metals influence Hg bioremediation dynamics.

Thank you for this very relevant and insightful comment. **The use of multi-contaminated matrices provides a relevant methodological advantage, as it more closely simulates the natural conditions of polluted soils, where bacterial communities are commonly exposed to a complex mixture of heavy metal pollutants.**

**In this study, despite the elevated concentrations of iron (6504 mg/kg) and chromium (341.03 mg/kg) detected in the soil,** Pseudomonas stutzeri **LBR exhibited consistent efficiency in mercury (Hg) bioremediation.**

**This ability is likely related to the strain's multimetal resistance profile. Previous studies have reported that** P. stutzeri **is resistant to chromium** [1] **(reference to be inserted) and capable of producing siderophores that bind iron, thereby reducing its bioavailability and potential toxicity** [2] **(reference to be inserted).**

**As noted by Nanda et al. (2019), bacterial strains tolerant to multiple metals are well suited for bioremediation purposes, as they tend to show better survival and functional performance in environments with complex metal contamination** [3]**.**

Here is the sentence included in the revised manuscript at Line 384:

“In the presence of elevated concentrations of iron (6,504 mg/kg) and chromium (341.03 mg/kg) in the soil, Pseudomonas stutzeri LBR maintained efficient Hg bioremediation, likely supported by its intrinsic multimetal resistance mechanisms [1,2]. The simultaneous presence of multiple background metals may promote bacterial adaptive responses, aligning the experimental setting more closely with the environmental complexity of real-world contaminated soils [3].”

1.	Kumari D, Pan X, Zhang D, Zhao C, Al-Misned FA, Mortuza MGolam. Bioreduction of Hexavalent Chromium from Soil Column Leachate by *Pseudomonas stutzeri*. Bioremediation J. 2015;19: 249–258. doi:10.1080/10889868.2015.1029116

2.	Essén SA, Johnsson A, Bylund D, Pedersen K, Lundström US. Siderophore Production by *Pseudomonas stutzeri* under Aerobic and Anaerobic Conditions. Appl Environ Microbiol. 2007;73: 5857–5864. doi:10.1128/AEM.00072-07

3.	Nanda M, Kumar V, Sharma DK. Multimetal tolerance mechanisms in bacteria: The resistance strategies acquired by bacteria that can be exploited to ‘clean-up’ heavy metal contaminants from water. Aquat Toxicol. 2019;212: 1–10. doi:10.1016/j.aquatox.2019.04.011

3. Page 14, Lines 298–302: o Issue: Bacterial counts use "10º" (e.g., 27 x 10º CFU/mL) instead of "10⁹". o Correction: Replace "10º" with "10⁹" throughout.

Thank you for your comment. The correct notation (e.g., 10⁹) was already used in the manuscript, but it may have appeared unclear due to font size or formatting. We have increased the font size of the superscript to improve legibility throughout the text. (Line 296)

4. Page 16, Line 345: o Issue: Acknowledgments list collaborators but omit funding sources. o Recommendation: Declare funding or state "No funding was received."

Thank you for your comment. We have added the following sentence to the Funding section:“The authors received no specific funding for this work.” At line 474.

**Data Availability (Throughout)**
1.Raw data (bacterial counts, Hg measurements) are not deposited in a public repository.
Action: Add a Data Availability Statement (e.g., "Data are available in [repository name] at [DOI/link]").

The raw data supporting the findings of this study, including mercury concentrations and bacterial counts, have been deposited in the Zenodo repository under the title ‘Raw data on mercury concentrations and bacterial counts’, and are accessible via DOI: 10.5281/zenodo.16621459. Available online from August 30, 2025 (at line 478 in revised manuscript)

2. GenBank Accession (Page 6, Line 125)
The *P. stutzeri* LBR sequence is cited as deposited in GenBank, but the accession number is missing.
Action: Provide the GenBank accession number in the Methods.

Thank you for your comment. The GenBank accession number is already provided in the methods on page 8, line 178.

Waiting for your reply,

My thanks in advance,

Dr Chiraz ABBES
