# Supplementary material for: Novel method for combining microbial bioremediation with static magnetic fields to remediate mercury-contaminated soils
Source: PLoS One. 2025 Aug 22;20(8):e0330872. doi: 10.1371/journal.pone.0330872 (PMC12373231; doi:10.1371/journal.pone.0330872)
Supplement: Attachment — Submitted filename: Reviewer comments.docx [file pone.0330872.s004.docx]

**Reviewer comments:**

Editor of PLOS ONE

I want to say thank you for giving me the opportunity to review the manuscript. I have read the present manuscript authored by Naima Werfelli and colleagues with keen interest and in detail. This manuscript explores a novel approach for enhancing microbial bioremediation by incorporating static magnetic fields to address mercury contamination in soils. Mercury, a persistent environmental pollutant, poses significant threats to ecosystems and human health.

Here are some comments and suggestions for improving the article:

1. Consider reorganizing the content to progress from general mercury contamination issues to the specific focus of your study. For example:
   - Mercury as an environmental pollutant (current impacts and sources).
   - Current remediation challenges (limitations of traditional methods).
   - Potential of bioremediation (your approach and objectives).
2. Some sentences are lengthy and contain multiple ideas. Consider breaking them down to improve clarity. For example: You mention that *"a variety of bacteria have the capacity to resist toxic forms of mercury and subsequently convert them into non-toxic forms."*
   - It would be helpful to briefly explain the mechanisms involved in these conversions.
   - You could also mention if P. stutzeri LBR has been compared with other strains for effectiveness.
3. The study objective is stated clearly at the end, but consider emphasizing what practical applications or next steps could follow from this research.
4. The material and method section is well-structured, but it could benefit from clearer subheadings and a more logical flow. Consider structuring it as follows for better readability:
   - Soil Sampling and Preparation
   - Soil Analysis (Physical, Chemical, and Heavy Metal Concentration)
   - Bacterial Strain and Inoculation
   - Bioaugmentation Tests
   - Static Magnetic Field (SMF) Application
   - Monitoring and Analysis
   - Statistical Analysis
5. Specify if the mercury solution was mixed uniformly to ensure even distribution in the soil.
   - Clarify why pH 7 was chosen and its significance in mercury solubility/stability.
   - Indicate whether control groups were included for comparative purposes.
   - The bacterial inoculum concentration (70 x 10⁹ cells) should mention if it was determined through OD measurements or CFU counting for better reproducibility.
   - Mention if post-hoc tests were applied to detect significant differences between groups.
   - Instead of "significance level of p < 0.05," consider rephrasing to "statistical significance was set at p < 0.05."
6. Ensure correct citation formatting, e.g.,
   - "[4 , 5]" should be "[4,5]" (remove spaces before commas).
   - Proper reference order for standards like "AFNORNF ISO 11265" should follow a uniform style.
   - Some paragraphs contain excessive detail; simplifying them can make the section more reader-friendly.
   - • Increase the clarity of tables and charts.
7. Summarize the key findings more clearly, highlighting the effectiveness of bioaugmentation and SMF's impact.
8. When presenting comparative results, such as the 49.36% and 72.49% remediation rates, consider briefly explaining why sterile soils showed a higher improvement compared to non-sterile soils. This helps provide context for the reader.
9. Clarify mechanisms where necessary; for instance, when discussing microbial competition in non-sterile soils, elaborate briefly on how this impacts mercury uptake.
10. Instead of saying "published studies," specify which studies to strengthen credibility.
11. A brief mention of potential future experiments to confirm these mechanisms could enhance the scientific rigor of the document.
12. Expand on future research opportunities, such as optimizing SMF parameters (intensity, duration), exploring its application in real-world contaminated sites, and assessing long-term environmental impacts.
13. Ensure uniform formatting in terms of punctuation, spaces, and abbreviations.
14. For example, journal names should follow a consistent format (e.g., *Atmos. Environ.* or *Atmospheric Environment*, but not a mix of both).

- Instead of writing the DOI as a URL, it's better to present it in the standard format: doi:10.xxxx/xxxx.
- Example:
  https://doi.org/10.1016/S1352-2310(97)00293-8 → doi:10.1016/S1352-2310(97)00293-8
- Ensure a consistent style for author initials (e.g., "W.H. Schroeder" vs. "WH Schroeder").
- Remove unnecessary spaces between initials.
- Ensure all references have complete citation details, such as volume, issue, page numbers, and publication year.
